# Polymeric Gelatin Scaffolds Affect Mesenchymal Stem Cell Differentiation and Its Diverse Applications in Tissue Engineering

**DOI:** 10.3390/ijms21228632

**Published:** 2020-11-16

**Authors:** Chia-Yu Wang, Po-Da Hong, Ding-Han Wang, Juin-Hong Cherng, Shu-Jen Chang, Cheng-Che Liu, Tong-Jing Fang, Yi-Wen Wang

**Affiliations:** 1 Department of Materials Sciences and Engineering, National Taiwan University of Science and Technology, Taipei 106, Taiwan; yu590909@gmail.com (C.-Y.W.); poda@mail.ntust.edu.tw (P.-D.H.); 2 Department of Dentistry, School of Dentistry, National Yang-Ming University, Taipei 112, Taiwan; nn2399906@ym.edu.tw; 3 Laboratory of Adult Stem Cell and Tissue Regeneration, National Defense Medical Center, Taipei 114, Taiwan; i72bbb@gmail.com (J.-H.C.); belle661011@gmail.com (S.-J.C.); 4 Department and Graduate Institute of Biology and Anatomy, National Defense Medical Center, Taipei 114, Taiwan; 5 Department of Gerontological Health Care, National Taipei University of Nursing and Health Sciences, Taipei 112, Taiwan; 6 Department of Physiology and Biophysics, Graduate Institute of Physiology, National Defense Medical Center, Taipei 114, Taiwan; chencheliu2002@gmail.com (C.-C.L.); wonderfulactioncom@gmail.com (T.-J.F.)

**Keywords:** gelatin, scaffold, human adipose stem cells, tissue regeneration, mesoderm lineage-specific differentiation

## Abstract

Studies using polymeric scaffolds for various biomedical applications, such as tissue engineering, implants and medical substitutes, and drug delivery systems, have attempted to identify suitable material for tissue regeneration. This study aimed to investigate the biocompatibility and effectiveness of a gelatin scaffold seeded with human adipose stem cells (hASCs), including physical characteristics, multilineage differentiation in vitro, and osteogenic potential, in a rat model of a calvarial bone defect and to optimize its design. This functionalized scaffold comprised gelatin-hASCs layers to improve their efficacy in various biomedical applications. The gelatin scaffold exhibited excellent biocompatibility in vitro after two weeks of implantation. Furthermore, the gelatin scaffold supported and specifically regulated the proliferation and osteogenic and chondrogenic differentiation of hASCs, respectively. After 12 weeks of implantation, upon treatment with the gelatin-hASCs scaffold, the calvarial bone harboring the critical defect regenerated better and displayed greater osteogenic potential without any damage to the surrounding tissues compared to the untreated bone defect. These findings suggest that the present gelatin scaffold is a good potential carrier for stem cells in various tissue engineering applications.

## 1. Introduction

Owing to a reduced supply and concomitantly increased demand for organ transplants, tissue engineering approaches offer hope to patients who are urgently in need of tissue and organ replacements. Since 1980, scientists have developed a novel method to generate complex structures with polymers, which exhibit the desired properties for specific tissue engineering applications. Polymer scaffolds may provide mechanical strength, interconnected porosity and surface area, varying surface chemistry, and unique geometry for direct tissue regeneration [1], for regenerating specific tissues or organs. Scaffolding is central to this effort, serving as a three-dimensional (3D) template for tissue ingrowth by mimicking the ECM [2].

Gelatin is a combination of a naturally occurring protein obtained from acidic and alkaline hydrolysis of collagen under a temperature- and pH-controlled environment [3,4,5,6]. Furthermore, gelatin is a protein-based polymer or biopolymer generated through thermal denaturalization of collagen [7]. Gelatin has advantageous features, including low budget, biological abundance, ease of production, and amplifying effects on cell proliferation [8]. Furthermore, gelatin is widely used as a biomaterial owing to its biocompatibility, non-immunogenicity, and potential for amino acid substitutions [4,9,10,11]. The RGD sequences (Arg–Gly–Asp) in gelatin facilitate initial cell adhesion [12]. Gelatin scaffold provide a suitable environment for bone reconstruction; however, it was degraded faster than desire primarily due to the gelatin component were enzymatically catalyzed hydrolysis in a living organism [13]. Polycaprolactone (PCL), a well-known polyester material, is primarily used to produce 3D scaffolding for tissue engineering applications. Its advantages include a relatively slow degradation rate and lower acidic by-products compared to other polyesters [14]. To enhance the mechanical properties and extend the degradation time of the scaffold, composite materials with PCL were used for various tissue regeneration applications [15]. Furthermore, studies have reported that cells in porous PCL scaffolding can retain exactly the same phenotype as chondrocytes in native cartilage tissue [16]. Taking together, the gelatin PCL-composite scaffold for tissue engineering has potential ability to mimic the native bone tissue environment.

The unusual potential of mesenchymal stem cells (MSCs) to modulate immune reactions contributes to their effective application in regenerative medicine and tissue engineering. Studies on numerous artificial extracellular matrices (ECMs) with different composites and their 3D distribution patterns are important to improve their therapeutic potentials [17]. The exposed microenvironment of 3D cultures, which mimics in vivo conditions, facilitates more efficient interactions between cells and their microenvironment [4]. Considering that the proliferation and differentiation of adult stem cells depends on various signals from their microenvironment, improvements have been made to the spread and differentiation of MSCs. Besides the choice of relevant material, cells are a fundamental concern in tissue engineering, such as bone tissue engineering (BTE) [18], cartilage tissue engineering (CTE) [19], and neural tissue engineering (NTE) [20]. MSCs responsible for maintaining bones, cartilage, muscle, and adipose tissue is capable of tissue regeneration, such as bone defect caused by fracture and any other injuries. MSCs have several excellent characteristics, which render them are widely used in tissue engineering approaches for therapeutic applications; these include their potential for self-renewal, convenience of segregation, expansion, and culturing in vitro and in vivo, and multilineage differentiation, e.g., adipocytes, chondrocytes, and osteoblasts [21,22,23,24,25]. MSCs can be obtained from several regions in the body, including adipose tissue, being referred to as human adipose stem cells (hASCs) [26]. Multilineage differentiation of hASCs with less intrusive accessibility renders them an effective candidate for cell therapy [27,28]. Previous studies culturing hASCs in various types of scaffolds have reported remarkable findings not only for osteogenesis, but also for other tissue generation, such as epithelialization, neurogenesis, and chondrogenesis [18]. Moreover, culturing of hASCs in scaffolds shortened the duration for wound healing [29,30,31].

Over the past few years, 3D bio-scaffolds have been developed through several methods including freeze-drying, phase separation, foam templating, electrospinning, melt processing, or solvent casting [32]. To enhance the final properties of the scaffold, the design strategy to effectively provide optimal treatments need to be considered. In this study, we produced a novel freeze-drying gelatin scaffold with PCL coating, and then the hASCs were seeded in it. The functional construct was designed by combining layers of the hASCs-gelatin scaffold with a convenient shape for handling. Our hypotheses are that the functionalized gelatin scaffold is biocompatible and able to regulate the hASCs proliferation and differentiation to optimize bone healing process. The effectiveness of the hASCs-gelatin scaffold for in vitro and in vivo bone regeneration was then evaluated.

## 2. Results

### 2.1. Characterization of the Stemness and Multilineage Differentiation of hASCs

**Figure 1:**
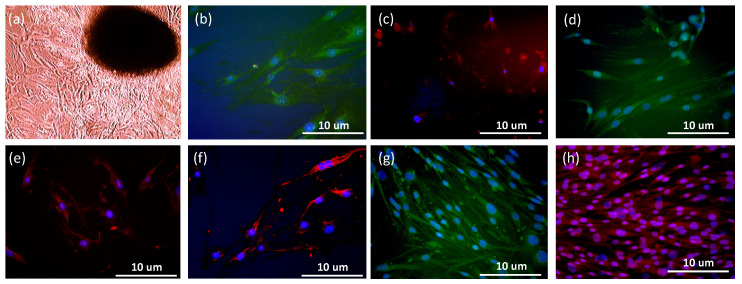
Characterization of the stemness and multilineage differentiation of human adipose stem cells (hASCs). The stemness of hASCs was characterized on the basis of several aspects: (**a**) sphere formation ability; (**b**) OCT-4 expression; (**c**) Nestin expression; (**d**) SOX-2 expression. However, multilineage differentiation of hASCs was assessed on the basis of the expression of the following markers: (**e**) NACM; (**f**) p75NTR; (**g**) Osteocalcin; (**h**) SOX-9. Nuclei counterstained with Hoechst 33342 are indicated in blue.

Figure 1 shows the characterization of hASCs used in this study. The sphere-forming potential of hASCs (Figure 1a) and positive expression of octamer-binding protein 4 (OCT-4; Figure 1b), nestin (Figure 1c), and SRY-box transcription factor 2 (SOX-2; Figure 1d) confirmed the stemness characteristics of hASCs. Further, to examine the differentiation potential of hASCs, immunostaining analysis with neural cell adhesion molecule (NACM; Figure 1e), p75 neurotrophin (p75NTR; Figure 1f), osteocalcin (Figure 1g), and SOX-9 (Figure 1h) antibodies as markers of neurogenesis, osteogenesis, and chondrogenesis, respectively, was performed, and all markers displayed positive expression.

### 2.2. Physical Characteristics and In Vitro Biocompatibility of the Gelatin Scaffold

**Figure 2:**
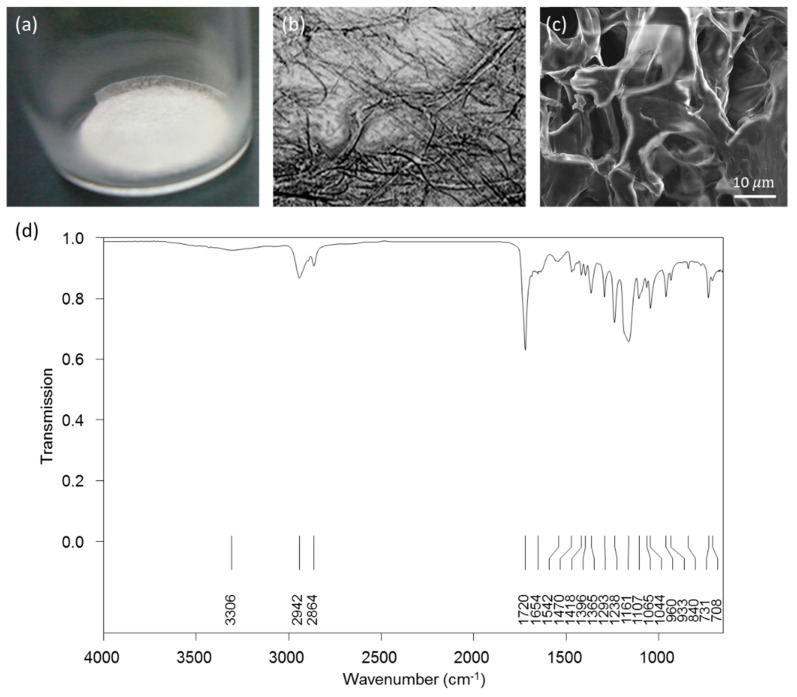
The morphology and characteristics of the gelatin scaffold. (**a**–**c**) Appearance of the scaffold upon macroscopic examination, visible light microscopy, and scanning electron microscopy, respectively; (**d**) Fourier transform infrared spectroscopic analysis.

The morphology of the gelatin scaffold with respect to its macroscopic appearance and on microscopic assessment are provided in Figure 2a,b, respectively. On scanning electron microscopy (SEM), the gelatin scaffold displayed a spongy structure (Figure 2c). Further, the structure of the gelatin scaffold was examined through Fourier transform infrared (FTIR) spectroscopy. Several major peaks were observed, including 2942 cm^−1^ and 1720 cm^−1^, corresponding to asymmetric CH_2_ and carbonyl stretching, respectively, and 1654 cm^−1^ (amide I), 1542 cm^−1^ (amide II), and 1238 cm^−1^ (amide III) as common bands of gelatin corresponding to the stretching of C–O, N–H, and C–N bonds, respectively (Figure 2d).

**Figure 3:**
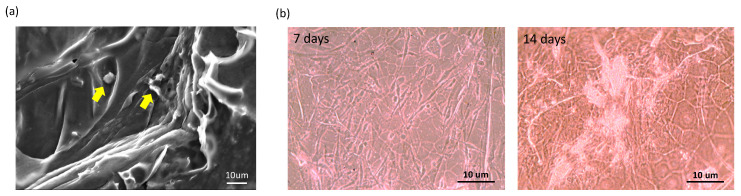
In vitro biocompatibility of human adipose stem cells (hASCs) seeded on the gelatin scaffold. (**a**) Scanning electron microscopy (yellow arrow: hASCs); (**b**) the growth of hASCs with time.

To evaluate the effect of the gelatin scaffold on the cellular microenvironment, we further incubated hASCs on the gelatin scaffold for seven and 14 days. Consequently, hASCs adhered well on the gelatin scaffold (Figure 3a) and proliferated well with time (Figure 3b).

### 2.3. Multilineage Differentiation of hASCs on the Gelatin Scaffold

**Figure 4:**
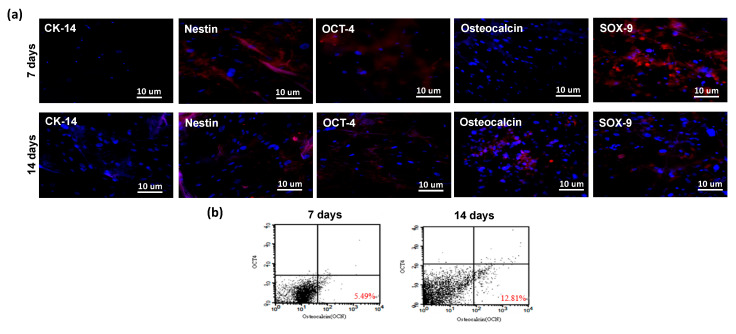
Immunofluorescence staining of human adipose stem cells (hASCs) cultured on the gelatin scaffold for 7 and 14 d. Nuclei counterstained with Hoechst 33342 are indicated in blue. (**a**) Multilineage differentiation of hASCs; (**b**) flow cytometric analysis of the osteocalcin marker.

**Figure 5:**
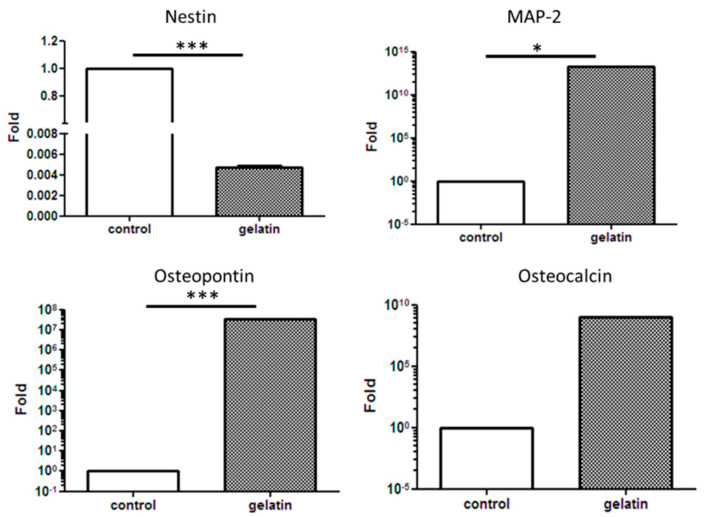
Analysis of the gene expression of nestin, microtubule-associated protein 2 (MAP-2), osteopontin, and osteocalcin in human adipose stem cells cultured on the gelatin scaffold. Data represent mean ± S. D., * *P* < 0.05, *** *P* <0.001 compared with control group.

**Figure 6:**
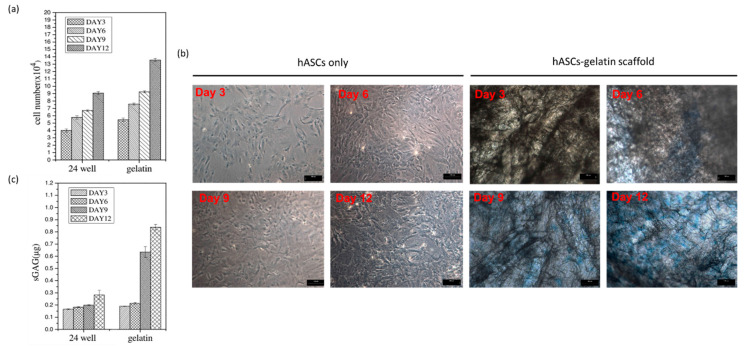
Evaluation of chondrogenesis of human adipose stem cells (hASCs) cultured on the gelatin scaffold. (**a**) Proliferation of hASCs; (**b**) Alcian Blue staining of the proteoglycan aggrecan, a chondrogenesis indicator, revealing dark-blue puncta (scale bar = 100 µm); (**c**) Dimethylmethylene (DMMB) Blue assay.

**Figure 7:**
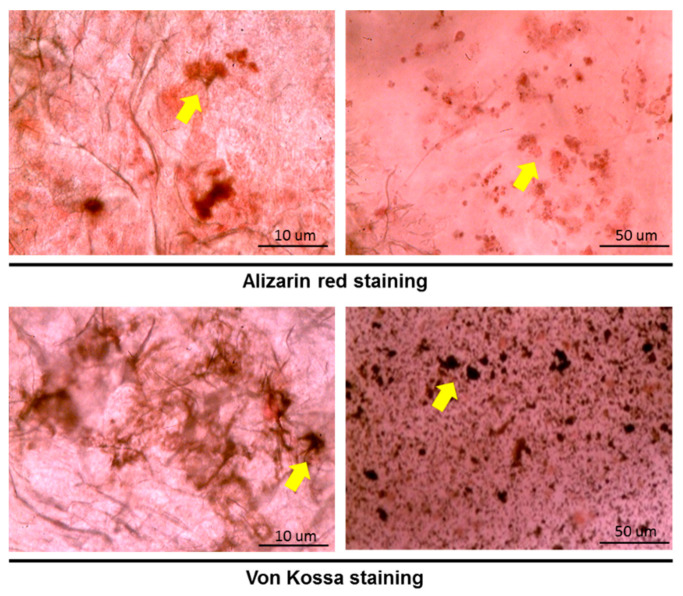
Evaluation of the mineralization of human adipose stem cells cultured on the gelatin scaffold for 14 days upon Alizarin Red and Von Kossa staining. Yellow arrows indicate calcium or phosphate deposition.

The effect of the gelatin scaffold on the differentiation of hASCs was examined using several differentiation markers including cytokeratin 14 (CK-14), OCT-4, nestin, microtubule-associated protein 2 (MAP-2), osteopontin, osteocalcin, and SOX-9, which are epidermal, epithelial, neural, osteogenic, and chondrogenic differentiation markers, respectively. Immunofluorescence staining revealed that nestin, OCT-4, and SOX-9 on the hASC-gelatin scaffold were downregulated after 14 days of incubation, whereas osteocalcin was upregulated (Figure 4a), implying that the gelatin scaffold specifically enhanced the osteogenic differentiation of hASCs. Flow cytometric analysis revealed that the density of osteocalcin-expressing cells increased from 5.49% to 12.81% with time (Figure 4b). Furthermore, gene expression analysis revealed that nestin was significantly downregulated on the hASC-gelatin scaffold, whereas MAP-2, osteopontin, and osteocalcin were upregulated in hASCs cultured on the scaffold compared to those cultured without the scaffold (Figure 5), indicating that the gelatin scaffold can induce mesoderm lineage-specific differentiation. Specifically, in chondrogenesis induction medium, the gelatin scaffold promoted hASC proliferation and displayed a positive signal on Alcian Blue staining (Figure 6a,b), indicating that the gelatin scaffold induced hASC differentiation to form a mature ECM. The DMMB assay further confirmed that levels of sulfated glycosaminoglycan (sGAG) in the hASC-gelatin scaffold were higher than those in the hASCs only with time (Figure 6c). Furthermore, the mineralization potential of hASCs seeded in the gelatin scaffold were examined through Alizarin Red and Von Kossa staining. Consequently, a bunch of red or black nodules representing calcium or phosphate deposition, respectively, were intensely observed (Figure 7).

### 2.4. In Vivo Assessment Using a Rat Calvarial Defect Model

**Figure 8:**
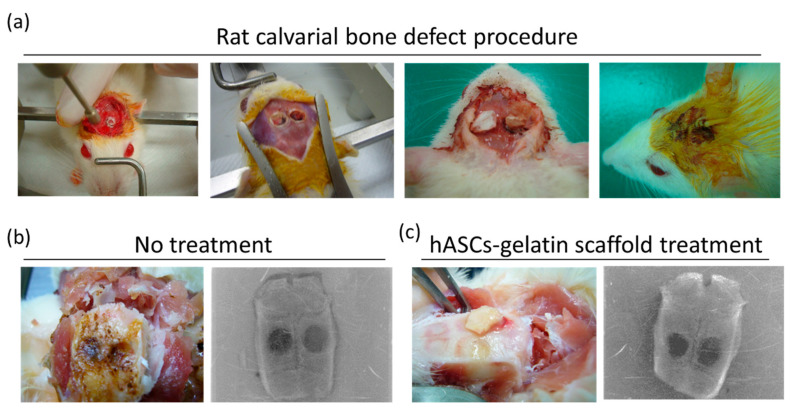
Rat model of calvarial bone defect. (**a**) Generation of the calvarial bone defect; (**b**) calvarial bone defect not treated with the hASC-gelatin scaffold at 12 weeks; (**c**) rat calvarial bone defect treated with the hASC-gelatin scaffold at 12 weeks of implantation.

The hASC-gelatin scaffold was implanted in the calvarial bone perforated with defects of 5-mm diameter in rats (Figure 8a) and compared to defects without scaffold treatment. After 12 weeks of implantation, the wound surface smoothly healed in the hASC-gelatin scaffold-treated group compared to that in the untreated group (Figure 8b,c). X-ray imaging revealed that the bone defect in the untreated group seemed completely spherical with almost no osteogenesis (Figure 8b), whereas the hASCs-gelatin scaffold group displayed irregular osteogenesis around the defect perforations (Figure 8c).

## 3. Discussion

**Figure 9:**
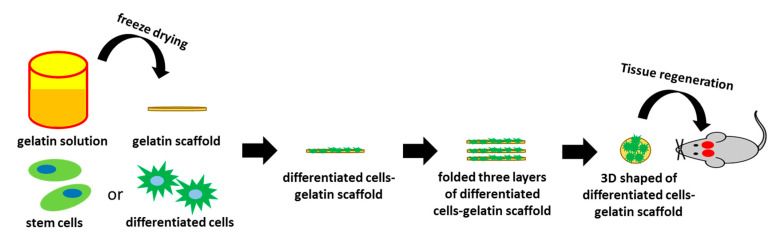
Schematic representation of the implantation of the functionalized gelatin scaffold for bone tissue regeneration in rats.

Several types of biomaterial have been extensively investigated for BTE [33,34]; however, designing of an optimal and effective bone substitute for defects has remained challenging. An important strategy for the generation of bio-scaffolds for BTE is the potential for mimicking an osteogenic ECM [35]. This study is aimed to investigate the potential effect of gelatin as a functional scaffold to assess cellular behavior during treatments aimed at bone healing. We developed a novel functionalized gelatin scaffold by combining layers of a hASCs-gelatin scaffold, that is easy to handle, to fill bone defects as desired (Figure 9). Owing to the spongy microstructure and unchanging structure of gelatin during scaffold preparation, the scaffold presented an outstanding microenvironment for cell proliferation and differentiation. The advantage of this scaffold design is that it effectively provided an appropriate ECM with abundant live cells into the defect area. ECM plays a key role in the stem cell niche to trigger various significant effects on physiological homeostasis and regeneration due to its directly or indirectly modulation of the self-renewal, and the differentiation of stem cells under disease conditions. There are two novelties of this study: we utilize early stage stem cells from a small amount of post-surgery medical waste (about 1–2 mL of adipose tissue) for experimental (Figure 1), and we find the optimal condition of freeze-drying method for gelatin with PCL coating scaffold fabrication (Figure 2) to solve the limitation of natural ECM bio-scaffold application in damaged tissue due to their poor mechanical strength property and uncontrollable degradation rates.

MTT cell proliferation assay is a well published method for the investigation of the cytotoxicity of a specific material [36]. Our results demonstrated that hASCs adhered well on the gelatin scaffold and greatly proliferated as time progressed in any medium condition (Figure 3 and Figure 6a), implying the absence of cytotoxic effect of gelatin on cell behavior. In line with our previous studies, gelatin scaffold after cultured with stem cells emerged as an effective regenerating biomaterial [37,38]. As a protein-based material, gelatin has excellent biocompatibility and low antigenicity and facilitates adequate cell adhesion owing to its interactions with cell surfaces [39,40]. When the cell adhesion proceeded and thus cell’s shape changed via cytoskeletal protein mediation, these reactions were favorable to the completion of the mitosis process [41,42]. Furthermore, gelatin reportedly influences stem cell differentiation [43]. Concurrent with previous reports [44,45], our results indicate that the gelatin scaffold specifically facilitated the osteogenic differentiation of hASCs, as revealed through osteocalcin upregulation; thus, is an ideal candidate material for BTE (Figure 4b and Figure 7). Furthermore, gene expression analysis revealed upregulation of MAP-2, osteopontin, and osteocalcin in hASCs cultured on the gelatin scaffold without medium induction (Figure 5). Gelatin combined with PCL reportedly serves as a positive cue supporting neurite outgrowth and facilitates neuronal differentiation in vitro, simultaneously supporting axon extension and concomitant MAP-2 expression [46]. Moreover, osteopontin and osteocalcin are late osteogenic markers secreted by mature osteoblasts, which indicate terminal osteoblast differentiation [47,48]. Although hASCs are mesodermal origin, they can differentiate into cells of ectodermal, endodermal, and mesodermal origin [49]. hASCs differentiation in vitro is induced by selective media containing lineage-specific induction factors [28]. However, this study is the first, to our knowledge, to report that gelatin biomaterials induce mesodermal lineage-specific differentiation among hASCs without a selection induction medium (Figure 4a). Nevertheless, further studies are required to investigate the mechanism underlying this phenomenon.

In this study, X-ray imaging revealed osteogenesis and adequate formation of surrounding tissues in the hASCs-gelatin scaffold group, whereas a completely spherical morphology of bone defects with almost no osteogenesis bone formation was observed in the untreated defect group (Figure 8b,c). Besides osteopontin and osteocalcin expression as bone differentiation markers, this study shows that hASCs seeded on the gelatin scaffold enhanced the GAG content (Figure 6b,c), implying its importance in the regeneration of mature cartilage ECM [50]. Chondrogenesis and osteogenesis occur synchronously in one continuous developmental phenomenon during endochondral osteogenesis [51]. Hence, both direct transformation of chondrocytes to osteoblasts and osteocytes occurred in hASCs seeded on the gelatin scaffold, being affected by focal adhesion induced by the gelatin molecules. When cells determine ECM protein patterns, a signaling pathway triggers the clustering of an integrin receptor at the plasma membrane and focal adhesion-associated protein recruitment, resulting in the maturing of the adhesion sites and mineralized ECM synthesis as an indicator of osteo-specific differentiation and osteoneogenesis [52,53]. Further, Kim et al. reported that gelatin positively influences early cell matrix formation and markedly induces cell proliferation and outstanding osteogenic differentiation of cells by activating the Wnt pathway and interacting with RGD sequences [44]. The present in vivo findings suggest that considering the material and design of the scaffold fabricated herein, it can effectively induce osteogenic differentiation of hASCs and serve as a promising strategy for BTE. In addition to BTE, this study shows that hASCs seeded in the gelatin scaffold are potentially applicable in CTE and NTE during organ transplantation. Altogether, our study results confirm that gelatin scaffold can be a cell carrier for functional tissue engineering, and also drive new challenges for producing biomaterial in tailored regenerative medicine.

## 4. Materials and Methods

### 4.1. Preparation of the Gelatin Scaffold

Gelatin (G9382, Sigma-Aldrich, Saint Louis, MO, USA) was dissolved in 1% acetic acid using a magnetic stirrer at 27 °C for getting gelatin solution (0.25% *w*/*v*). The glass vial was then used for depositing 0.275 mL of aliquots of the gelatin solution, which were then frozen at −20 °C for approximately 1 h. Thereafter, the frozen gelatin solution was moved to a freeze dryer (FD24-4S; Kingming, Taipei, Taiwan, R.O.C.) at −45 °C and 300 mbar vacuum condition. After freezing for 24 h, the gelatin mats were treated with 0.5 mL of 2.5% *w*/*v* PCL/dichloromethane solution to obtain 1:20 *w*/*v* gelatin biocomposites. Further, the glass vial was closed for 30 min before finally opening the vial lid for solvent evaporation overnight.

### 4.2. Physical Characterization of the Gelatin Scaffold

#### 4.2.1. SEM Assessment

Firstly, the sample was sectioned, attached to the carbon stubs, and then coated with gold by using a sputter coating machine. The SEM analysis was performed using the HITACHI S-3000N (Hitachi High Technologies, Krefeld, Germany). SEM images were obtained at an accelerating voltage of 1.5 kV.

#### 4.2.2. FTIR Spectroscopy

The structural of gelatin scaffold was analyzed via FTIR spectroscopy on a Nicolet 8700 Research spectrometer (Thermo Scientific, Waltham, MA, USA). The resolution of 32 scans was 4 cm^−1^ and the wavenumber range was from 400 cm^−1^ up to 4000 cm^−1^ for each measurement.

### 4.3. Coculture of Human Adult Adipose Stem Cells (hASCs)

ASCs from freshly harvested surgical waste were isolated using a previously described method [54]. Briefly, tissue samples were obtained as certified by the Institutional Review Board of Tri-Service General Hospital, Taipei, Taiwan, R.O.C. (IRB approval number: 2-105-05-150). The adipose tissues were processed become small pieces and then incubated with digesting medium in DMEM (Gibco-Invitrogen, Carlsbad, CA, USA) containing 1% penicillin/streptomycin (Merck, Darmstadt, Germany), 0.1% glucose Ferak, Berlin, Germany), and 0.1% collagenase (Sigma-Aldrich, St-Louis, MO, USA) at 37 °C in 5% CO_2_ for 24–36 h according to the cells attachment. The primary culture of ASCs (supplied by Dr. Cherng) were cultured in a keratinocyte serum-free medium (KSFM; Life Technologies Ltd., Paisley, Scotland, UK), antioxidants N-acetyl-cysteine and L-ascorbic acid-2-phosphate (Sigma, St. Louis, MO, USA) supplemented with a 10% fetal bovine serum (FBS; Hyclone, Logan, UT, USA) in a 37 °C incubator with humidified air containing 5% CO_2_. To approach confluency, each generation lasted up to 2–3 days during their growth. The cells were then seeded at 5 × 10^4^ cells/well in 6-well plates for further evaluation.

#### 4.3.1. hASCs Seeding onto Gelatin Scaffold

The gelatin scaffolds were sterilized through UV irradiation for 18 h before use, followed by seeding of hASCs on the scaffolds at 4 × 10^5^ cells/cm^2^ and incubation in 5% CO_2_ at 37 °C. The culture medium was replaced up to 2–3 days during incubation.

#### 4.3.2. Assessment of Cell Viability

The viability of hASCs was assessed on days 3, 6, 9, and 12, through mitochondrial-dependent reduction of MTT to formazan. The 2.5 mg/mL of MTT reagent (Sigma-Aldrich; Merck, Darmstadt, Germany) in PBS was incubated with samples at 37 °C for 2 h. All samples were used in triplicate and the optical density was determined through automated dual-wavelength spectrophotometry (Bio-Tek ELX-800; BioTek, Winooski, VT, USA) against a reagent blank (i.e., no cells) at OD 570 nm. The cells were enumerated from a standard curve plotted using a known cell number.

#### 4.3.3. Assessment of the Multilineage Differentiation of hASCs and Immunofluorescence Staining

All well plates containing hASCs and hASCs-gelatin scaffolds were cultured in growth medium, keratinocyte induction medium, and osteocyte osteogenesis induction medium [37], chondrogenesis induction medium [38], and neurogenesis induction medium in accordance with our laboratory protocol. All well plates were then fixed with 4% paraformaldehyde, washed with phosphate-buffered saline (PBS), and permeabilized with 0.2% Triton X-100 (Sigma, St. Louis, MO, USA) for 15 min. Further, the samples were washed thrice with PBS, blocked with 10% normal goat serum (Gibco, Carlsbad, CA, USA), and incubated with a primary antibodies (OCT-4, CK-14, nestin, SOX-2, NACM, p75NTR, osteocalcin, SOX-9; all with 1:500 dilution, Santa Cruz Laboratories, Dallas, TX, USA) for 2 h at 37 °C. After the primary antibodies were washed with PBS, the samples were incubated with rhodamine- or fluorescein isothiocyanate (FITC)-conjugated secondary antibodies for 2 h at 37 °C. Hoechst 33342 staining (1:5000 dilution, AnaSpec, Fremont, CA, USA) was then performed for the sample for 15 min for visualizing the nuclei. Finally, fluorescence microscopic images were obtained using a fluorescent microscope (Axio Lab.A1, Carl Zeiss AG, Oberkochen, Germany) with a camera (Zeiss AxioCam ICm1, Carl Zeiss AG, Oberkochen, Germany).

#### 4.3.4. Flow Cytometry

hASCs were acquired using 0.25% trypsin-EDTA and FBS and then supplemented with PBS for 5 min with agitation. Further, hASCs were fixed with alcohol acetic acid solution (95% alcohol + 5% acetic acid) for 5 min. hASCs were then rinsed with PBS and with 0.05% NP-40 (diluted with PBS), the hASCs were blocked with 2% FBS for 10 min. Thereafter, hASCs were treated with the primary antibody and refrigerated at 4 °C overnight. Thereafter, hASCs were rinsed with PBS and incubated with rhodamine- or fluorescein isothiocyanate (FITC)-conjugated secondary antibodies for 2 h at 37 °C. After rinsing with PBS, the hASCs were fixed with 1% paraformaldehyde and flow cytometry was performed (BD FACSCalibur, BD Biosciences, Franklin Lakes, NJ, USA).

#### 4.3.5. Quantitative RT-PCR Analysis

Total RNA was extracted from the cells, using TRIzol (Invitrogen, Carlsbad, CA, USA) in accordance with the manufacturer’s instructions. Oligo (dT) primers were further used with reverse transcriptase (SuperScriptTM III RT; Invitrogen, Carlsbad, CA, USA) to synthesize cDNA from 1 μg of total RNA in accordance with the manufacturer’s protocol. Nestin, MAP-2, osteopontin, and osteocalcin gene expression levels were determined using real-time PCR. Target gene expression levels were then normalized to those of *GAPDH* levels, using the −ΔΔC^t^ method, and first-strand cDNA was subjected to real-time PCR using the LightCycler 480 Real-Time PCR System (Roche Applied Science, Penzberg, Germany). LightCycler 480 v1.5.0 was used to evaluate PCR kinetics and mRNA using the standard curve method.

#### 4.3.6. Alcian Blue Staining

Briefly, hASCs-gelatin scaffolds were fixed with a 0.1% acetic acid solution for 30 min, washed twice with PBS, and refrigerated at 4 °C for overnight. The samples were then immersed in the post-fixed solution containing 4% para-formaldehyde-sucrose for up to 3 d. Thereafter, the samples were rinsed with distilled water before staining with Alcian blue solution (Sigma-Aldrich; Merck, Darmstadt, Germany) for 5 min. The stained samples were then examined using a light microscope, and the images were obtained using a SPOT-RT digital camera (Diagnostic Instruments, Detroit, MI, USA).

#### 4.3.7. Quantification of Sulfated Glycosaminoglycan (sGAG) Content

hASCs-differentiated chondrocytes cultured on the gelatin scaffold were digested overnight in a digestion buffer (250 mg/mL papain (Sigma-Aldrich; Merck, Darmstadt, Germany), 0.2 M NaH_2_PO_4_, 0.1 M EDTA, 0.01 M cysteine) at 60 °C prior to quantification with the 1,9-dimethylmethylene blue (DMMB) sGAG assay. Different concentrations (0, 1, 2, 3, 4, 5, 6 μg/mL) of standard chondroitin-4-sulphate (C4S) stock solutions were prepared along with DMMB dye pipettes in 96 well microplate and the volume was adjusted to 20 μL with distilled water. A volume of 100 μL of each collected ASCs sample was pipetted in subsequent wells containing 500 μL of DMMB dye in the microplate, followed by agitation for 5 s, using a microplate shaker. Absorption was immediately recorded using the UV-visible spectrophotometer (UV 500, UNICAM) at 525 nm and plotted against the graph concentration and the CS content was determined from a standard graph.

### 4.4. Mineralization Evaluation

#### 4.4.1. Alizarin Red Staining

The hASCs-gelatin scaffolds were stained with Alizarin Red solution (Sigma-Aldrich; Merck, Darmstadt, Germany) for 5 min. Eventually, the staining liquid was removed, and the samples were washed thrice with distilled water. The stained samples were then examined using a light microscope, and the images were obtained with using a SPOT-RT digital camera (Diagnostic Instruments, Detroit, MI, USA).

#### 4.4.2. Von Kossa Staining

The hASCs-gelatin scaffolds were stained using a Von Kossa kit (Abcam, Cambridge, UK) in accordance with the manufacturer’s instructions. The stained samples were further examined using a light microscope, and the images were obtained using a SPOT-RT digital camera (Diagnostic Instruments, Detroit, MI, USA).

### 4.5. Rat Calvarial Defect Model

Eight-week-old male Sprague-Dawley rats were purchased from Bio-LASCO Co. Ltd. (Taipei, Taiwan, R.O.C.). The experimental protocol was approved by the Institutional Animal Care and Use Committee (IACUC-15-353) at the National Defense Medical Center (Taipei, Taiwan, R.O.C.). Eleven rats were divided into no treatment (n = 5) and hASCs-gelatin scaffold treatment (n = 6) groups. Prior to surgery, rats were anaesthetized through intraperitoneal injection of xylazine (8 mg/kg) and ketamine (100 mg/kg), and subcutaneous injection of enrofloxacin (0.05 mg/kg) as microbial prophylaxis. After exposing the calvarial bone, two bone defects with a 5-mm diameter were induced per cranium by using a trephine burr. The calvarial defects were either left empty as a no treatment group or surgically filled by inserting a hASCs-gelatin scaffold. After implantation, the injuries were sutured and extensively treated to prevent infection and damage to the dura mater. The rats were euthanized for 12 weeks post-implantation. Calvarial bone specimens were then harvested for further evaluation.

### 4.6. X-ray Imaging

The collected calvarial bone specimens were radiographically imaged using a microradiography unit (PY-70C, POYE, New Taipei City, Taiwan, R.O.C.) and scanned at 70 kV/10 mA source voltage/current with an X-ray film (FR; Fuji photo film, Tokyo, Japan) under standardized conditions for a 1.2-s exposure duration. Proper magnification was used during the observation and the micrograph results were compared among groups.

### 4.7. Statistical Analysis

Gene expression and sGAG levels are presented as mean ± SD values. Statistical analysis was performed using the Statistical Package for Social Science (SPSS, Chicago, IL, USA). The findings were considered statistically significant when *p* < 0.05. Student’s *t*-test was performed to assess significant differences in the study parameters between the two groups.

## 5. Conclusions

This study shows that the hASC-gelatin scaffold generated in this study has excellent biocompatibility in vitro and in vivo and directs the growth and specific osteogenic differentiation of hASCs, thus accelerating tissue regeneration at the calvarial bone defect in the present rat model without any tissue damage after implantation. Furthermore, this study shows that hASCs could be cultured in gelatin with or without a selected induction medium, indicating the potential of gelatin to induce mesodermal lineage-specific differentiation of hASCs. Together, the present results indicate that the present gelatin scaffold serves as a suitable carrier for stem cells in various tissue engineering applications.

## Abbreviations

BTE	Bone Tissue Engineering
PCL	Polycaprolactone
hASCs	Human Adipose Stem Cells
ECM	Extracellular Matrix
MSCs	Mesenchymal Stem Cells
CTE	Cartilage Tissue Engineering
NTE	Neural Tissue Engineering
MAP-2 OCT-4	Microtubule-Associated Protein 2 Octamer-Binding Protein 4
SOX-2	SRY-box Transcription Factor 2
SOX-9	SRY-box Transcription Factor 9
NACM	Neural Cell Adhesion Molecule
p75NTR	p75 Neurotrophin
CK-14	Cytokeratin 14
FBS	Fetal Bovine Serum
PBS	Phosphate-Buffered Saline
SEM	Scanning Electron Micrographs
FTIR	Fourier Transform Infrared
